# Apigenin directly interacts with and inhibits topoisomerase 1 to upregulate CD26/DPP4 on colorectal carcinoma cells

**DOI:** 10.3389/fphar.2022.1086894

**Published:** 2022-12-22

**Authors:** Julia E. Fux, Émilie C. Lefort, Praveen P. N. Rao, Jonathan Blay

**Affiliations:** ^1^ School of Pharmacy, University of Waterloo, Waterloo, ON, Canada; ^2^ Department of Pathology, Dalhousie University, Halifax, NS, Canada

**Keywords:** apigenin, DPP4 (CD26), CK2 (casein kinase II), topoisomerase 1 (Top1), irinotecan (CPT-11)

## Abstract

**Introduction:** CD26/dipeptidyl peptidase IV (DPP4) is a cell-surface glycoprotein present on most epithelial cells that modulates the local response to external signals. We have previously shown that the dietary flavone apigenin (4′,5,7-trihydroxyflavone) upregulates cell-surface CD26/DPP4 on human colorectal carcinoma (CRC) cells and regulates its activities. We observed a unique synergistic interaction with the CRC chemotherapeutic agent irinotecan, which through its metabolite SN38 elevates CD26 at doses that are sub-cytotoxic. As SN38 interacts with topoisomerase 1 (Topo1) we evaluated whether apigenin influences Topo1 activity.

**Methods:** We used a radioimmunoassay to selectively measure CD26 at the cell surface of HT-29 cells following various treatments. Topoisomerase 1 mRNA expression was measured by q-RT-PCR and protein abundance by western blot analysis. Direct inhibition of topoisomerase activity was measured using an assay of DNA supercoil relaxation with recombinant human Topo1. The role of Topo1 in the effect of apigenin was shown both pharmacologically and by siRNA silencing of Topo1. Molecular docking analysis was done with SBD computational software using the CDOCKER algorithm.

**Results:** The interplay between apigenin and irinotecan was not observed when apigenin was combined with other chemotherapeutic drugs including the topoisomerase 2 inhibitors doxorubicin or etoposide. There was no enhancement of irinotecan action if apigenin was replaced with its hydroxylated metabolite luteolin (3′,4′,5,7-tetrahydroxyflavone) or emodin (6-methyl-1,3,8-trihydroxyanthraquinone), which is an inhibitor of the principal kinase target of apigenin, casein kinase 2 (CK2). Apigenin did not alter Topo1 mRNA expression, but siRNA knockdown of functional Topo1 eliminated the effect of apigenin and itself increased CD26 levels. Apigenin inhibited Topo1 activity in intact HT-29 cells and showed comparable inhibition of purified recombinant human Topo1 enzyme activity to that of SN-38, the active metabolite of irinotecan. Apigenin fits into the complex of Topo1 with DNA to directly inhibit Topo1 enzyme activity.

**Discussion:** We conclude that apigenin has a unique fit into the Topo1-DNA functional complex that leads to direct inhibition of Topo1 activity, and suggest that this is the basis for the exceptional interaction with the CRC drug irinotecan. A combined action of these two agents may therefore exert a role to limit local signals that facilitate tumour progression.

## Introduction

Apigenin (4′,5,7-trihydroxyflavone; 5,7-dihydroxy-2-(4-hydroxyphenyl)-4H-1-benzopyran-4-one) is one of many bioactive compounds found in the diet, being present at significant levels in many fruits, vegetables, herbs, and spices and particularly abundant in parsley and chamomile (Birt et al., 2001; Yang et al., 2001; Lefort and Blay 2013). As a flavonoid, it has the potential to impact multiple cellular signalling pathways and consequent cell behaviour, giving it the capacity to alter the relationship of cells with their surroundings and potentially affect cell disposition in both normal and pathological situations. We have reviewed these actions and how they might impact on cancer, particularly in neoplasias situated in, or associated with, the gastrointestinal tract (Lefort and Blay 2013). Most importantly, we have recently shown that the use of oral supplements of apigenin in a semi-purified form should be able to reach circulating blood levels capable of causing the cellular changes that are predicted to be beneficial against cancers, including those of the gastrointestinal tract (DeRango-Adem and Blay 2021). This may be achieved using dosing quantities comparable with those currently accepted for other natural product supplements (DeRango-Adem and Blay 2021).

Amongst its actions, we have shown that apigenin is able to upregulate CD26 at the surface of human colorectal carcinoma (CRC) cells, including those of the HT-29, HRT-18 and Caco-2 cultured cell lines (Lefort and Blay 2011; Lefort et al., 2020). CD26 is a 110-kDa membrane glycoprotein that is normally found at the apical surface of mature intestinal epithelial cells including those of the colon (Darmoul et al., 1991; Lefort and Blay 2013). It shows changes in expression and distribution in cancer (Balis 1985; Kotackova et al., 2009). CD26 is a multifunctional protein and in its mature, dimeric form it is enzymatically active at the cell surface and has an intrinsic hydrolase activity (dipeptidyl peptidase IV, DPP4) that cleaves N-terminal dipeptides from certain extracellular signalling peptides (De Meester et al., 1999; Havre et al., 2008). Amongst its substrates that are key cell mediators are the cell-directing peptides known as chemokines, and the highest affinity of DPP4 is for CXCL12 (stromal-derived factor-1, SDF-1), which is the ligand for the receptor CXCR4, a regulator of cell migration that is itself regulated by constituents of the tumour microenvironment (Richard et al., 2006; Richard et al., 2007; Richard and Blay 2008). CD26 also (i) functions as the major cellular binding protein for adenosine deaminase (ADA), a soluble ecto-enzyme that inactivates the immunosuppressive metabolite adenosine (Dong et al., 1997), and (ii) directly interacts with the extracellular matrix (ECM) proteins collagen and fibronectin (Loster et al., 1995; Cheng et al., 2003). CD26 is therefore a nexus of regulation governing the cell’s relationship with its extracellular milieu, and it is regulated by apigenin.

We have recently shown (Lefort et al., 2020) that apigenin’s ability to upregulate CD26 is dependent upon its ability to inhibit the activity of casein kinase 2 (CK2). CK2 is a ubiquitous cellular Ser/Thr-protein kinase that has downstream effects on cell proliferation, cell viability, modulation of DNA damage, contractility, and cellular invasion through the extracellular matrix (Pinna and Meggio 1997; Morales and Carpenter 2004; Kim et al., 2018; Suhas et al., 2018). Apigenin is well known for its ability to inhibit CK2 and has been used experimentally as a CK2 inhibitor (Ahmad et al., 2006; Kroonen et al., 2012; Jung et al., 2014; Kim et al., 2018; Suhas et al., 2018). However, it is uncertain that apigenin exerts its effect on cellular behaviours including regulation of interactions with the extracellular matrix, cellular contractility and the function of molecules including CD26 (Lefort and Blay 2011; Kim et al., 2018; Suhas et al., 2018) solely through CK2. Apigenin and related flavonoids differ from synthetic CK2 inhibitors both in terms of the stereochemistry of their interaction with CK2 and their observed effect in experimental cellular systems (Sarno et al., 2002; Suhas et al., 2018). Furthermore, However, the protracted time course of the change in CD26 levels [maximum reached after 24 h–48 h, (Lefort and Blay 2011)] suggests that there are additional or subsequent events necessary in order to have changes in CD26.

In our earlier work (Lefort and Blay 2011) we noted that apigenin has a distinct property in how it interacts with the anti-cancer drug irinotecan at the cellular level. Multiple chemotherapeutic agents also have the property, independent of their cytotoxic action, of upregulating CD26 (Cutler et al., 2015). However while the effects of combining apigenin with, for example, 5-fluorouracil or oxaliplatin are simply additive, the combination with irinotecan shows a marked synergism, with irinotecan enhancing the potency for apigenin to increase CD26 by 30-fold (Lefort and Blay 2011). The primary mode of action of irinotecan, through its active metabolite SN-38, is to poison the nuclear enzyme DNA topoisomerase 1 (Topo1) (Liu et al., 2000). We therefore hypothesized that apigenin might somehow also be acting on Topo1, which would lead to an interaction with SN38 and explain the synergism with irinotecan in elevating CD26.

In this work we show that apigenin can directly interact with and inhibit the activity of topoisomerase, that the interaction is selective for Topo1, and that the resultant inhibition is a part of the ability of apigenin to upregulate CD26/DPP4.

## Materials and methods

### Culture of human colorectal carcinoma cells

HT-29 human colorectal carcinoma (CRC) cells were obtained from the American Type Culture Collection (ATCC, Manassas, VA, United States) and were maintained at 37°C in a humidified atmosphere of 90% air/10% CO_2_. The stocks used have been confirmed for genotype, and cultures were negative for *mycoplasma* contamination when tested using a PCR-based approach. All responses have been confirmed in cells within 4 passages of receipt from ATCC.

Cells were cultured in 80-cm^2^ flasks (Corning, Nepean, ON, Canada), containing Dulbecco’s modified Eagle medium (DMEM), supplemented with 10% (v/v) heat-inactivated newborn calf serum (NCS; Life technologies, Burlington, ON, Canada) and passaged with brief exposure to TrypLE™ Express (Life technologies, Burlington, ON, Canada). For experimental purposes, cells were seeded at density of 90,000 cells/ml unless otherwise indicated. Once cultures reached 60%–70% of confluent density, they were treated with compounds of interest or with control vehicle, as specified in figure legends.

Chemotherapeutic treatments were irinotecan (Sandoz, Montreal, QC, Canada), etoposide (Novopharm, Toronto, ON, Canada) and doxorubicin (Mayne Pharma, Montreal, QC, Canada). Bovine serum albumin (BSA), dimethyl sulfoxide (DMSO), apigenin, luteolin (3′,4′,5,7-tetrahydroxyflavone), 6-methyl-1,3,8-trihydroxyanthraquinone (emodin), and the irinotecan active metabolite SN-38 (7-Ethyl-10-hydroxycamptothecin) were obtained from Sigma-Aldrich (St. Louis, MO. United States). Flavonoids were dissolved in DMSO to make stock solutions and subsequently further diluted in medium to give a final DMSO concentration in whole cell assays of less than 0.02% (v/v), a concentration that does not affect CD26 levels (Tan et al., 2004).

### Radioimmunoassay for cell-surface CD26

Following a 48 h treatment, cell-surface CD26 was quantified using a radioimmunoassay as previously described (Tan et al., 2004). All washes and incubations were performed at 4°C. Wells from a 48- (or 24-) well plate were washed once with 500 µl (750 µl for 24-well plate) of ice-cold phosphate-buffered saline (PBS; 137 mM NaCl, 24.8 mM Tris-HCl, 5mM KCl, 0.7 mM Na_2_HPO_4_, 0.5 mM MgSO_4_ and 1 mM CaCl_2_; pH 7.2) containing 0.2% (w/v) BSA and incubated for 1 h with 125 µl (200 µl for a 24-well plate) PBS containing 1% (w/v) BSA and 1 μg/ml mouse anti-human CD26 (clone M-A261) monoclonal antibody (mAb) or mouse IgG isotype-matched control mAb (clone W3/25). Following the incubation period, cells were washed twice with 500 µl (750 µl for a 24-well plate) PBS containing 0.2% (w/v) BSA and were incubated for 1 h with 125 µl (200 µl for a 24-well plate) PBS containing 1% (w/v) BSA and 1 μCi/ml ^125^I-labeled goat anti-mouse IgG mAb obtained from (PerkinElmer Life Sciences, NEN, Boston, MA, United States). Cells received two final washes with 500 µl (750 µl for a 24-well plate) PBS containing 0.2% (^w^/_v_) BSA. Finally, 500 µl of 0.5 M NaOH was added to each well in order to solubilize the cells, and radioactivity was assessed using a gamma counter (Model 1,480 Wizard™ 3, Wallac Co., Turku, Finland). Radioactive counts were corrected for both non-specific binding relative to an isotype control and the number of viable cells as assessed by a Coulter^®^ Model ZM151183 particle counter (Beckman Coulter, Mississauga, ON, Canada).

### Topoisomerase 1 mRNA expression using q-RT-PCR

Total RNA was extracted from HT-29 cells grown in 6-well plates (VWR International, Mississauga, ON, Canada) using the TRIzol^®^ reagent (Life technologies, Burlington, ON, Canada), as indicated by the manufacturer. RNA concentrations were quantified by spectrophotometric analysis at 280 nm/260 nm wavelengths. RNA (1 μg) was reverse-transcribed using an M-MLV reverse transcriptase enzyme, 5 mM deoxyribonucleotide triphosphate, 0.5 µM oligo (dT), dithiothreitol, 5x First Strand buffer, all obtained from Life technologies (Burlington, ON, Canada) and DEPC-H_2_O (GE Healthcare Life Sciences, Baie D’Ufré, QC, Canada) in a total volume of 20 µl. Custom primers (Life technologies, Burlington, ON, Canada) were designed to amplify specific regions in the transcript and the sequences used were: Topo1 forward primer 5′- TCC​GGA​ACC​AGT​ATC​GAG​AAG​A-3′ and reverse primer 5′- CCT​CCT​TTT​CAT​TGC​CTG​CTC-3′. For each of the samples, mRNA levels were normalized to cyclophilin A, sequence forward: 5′-TTC​ATC​TGC​ACT​GCC​AAG​AC-3′, reverse: 5′-TCG​AGT​TGT​CCA​CAG​TCA​GC-3′. Amplification reactions included an initial cycle of denaturation for 10 min at 95°C, followed by 40 cycles of denaturation at 95°C for 20 s, annealing at 60°C for 18 s, and extension at 70°C for 30 s, with brilliant SYBR Green detection, and a final melting curve cycle of 95°C for 1 min, 65°C for 30 s, and 95°C for 30 s in a Stratagene Mx3000P thermocycler (Cedar Creek, TX, United States). Relative *Topo1* expression as indicated by the fluorescence was analyzed using the comparative Ct method.

### Western blot analysis for topoisomerase 1

HT-29 cells were seeded into 6-well plates and were grown to 60%–70% confluency, after which they were exposed to apigenin over a time period of 0 h–60 h. Cells were washed twice with ice-cold PBS and dissolved in lysis buffer (50 mM Tris-HCl, pH 7.4, 1% Nonidet P-40, 0.25% sodium deoxycholate, 150 mM NaCl) supplemented with 1 mM EDTA, 1 mM NaF, 1 mM phenylmethyl sulfonyl fluoride and 1X protease inhibitor cocktail set 1 (EMD Canada Inc. Mississauga, ON, Canada). Samples were incubated on a plate rotator for 20 min at 4°C and cell lysates then clarified by centrifugation (12,000 x *g* for 20 min). Cellular protein was quantified using Bio-Rad Protein Assay Dye Reagent (Bio-Rad Laboratories Inc. Mississauga, ON, Canada).

HT-29 cells were seeded into 6-well plates and were grown to 60%–70% confluency, after which they were exposed to apigenin over a time period of 0 h–60 h. Cells were washed twice with ice-cold PBS and dissolved in lysis buffer (50 mM Tris-HCl, pH 7.4, 1% Nonidet P-40, 0.25% sodium deoxycholate, 150 mM NaCl) supplemented with 1 mM EDTA, 1 mM NaF, 1 mM phenylmethyl sulfonyl fluoride and 1X protease inhibitor cocktail set 1 (EMD Canada Inc. Mississauga, ON, Canada). Samples were incubated on a plate rotator for 20 min at 4°C and cell lysates then clarified by centrifugation (12,000 x *g* for 20 min). Cellular protein was quantified using Bio-Rad Protein Assay Dye Reagent (Bio-Rad Laboratories Inc. Mississauga, ON, Canada). Samples were denatured in Laemmli buffer at 95°C for 5 min and 15 µg of protein per lane was separated by SDS-PAGE using 4% stacking and 10% resolving gels as previously described [27]. Gels were then electroblotted onto a nitrocellulose membrane, blocked with 3% BSA (CD26) and probed overnight at 4°C with IgM mouse anti-human DNA Topo1 (clone C-21) (BD Pharmingen, San Diego, CA, United States). Membranes were washed 5 times with Tris-buffered saline with 0.1% Tween 20 and then incubated with horseradish peroxidase (HRP)-conjugated polyclonal IgM goat anti-mouse secondary Ab (BD Pharmingen, San Diego, CA. United States), for 1 h at room temperature. Protein expression was detected using an enhanced chemiluminescence detection system (Thermo scientific, Ottawa, ON, Canada). To confirm equal protein loading in each sample, the membrane was re-probed with a IgG rabbit anti-human α–tubulin (11H10) primary Ab (Cell Signaling Technology^®^, Pickering, ON, Canada), followed by an IgG HRP-conjugated goat anti-rabbit secondary mAb (BD Pharmingen, San Diego, CA, United States).

### Assay of inhibition of recombinant topoisomerase 1

Recombinant human DNA topoisomerase 1 (rhTopo1; Prospec-Tany Technogene Ltd., East Brunswick, NJ, United States) was diluted to 10 μg/ml in 10 mM Tris-HCl, pH 7.9, 150 mM NaCl, 1 mM EDTA, 5% glycerol, 0.1 mM spermidine and 0.1% BSA, and used at 10 ng per reaction. The rhTopo1 was pre-incubated with flavonoids for 15min at room temperature, followed by incubation for 25 min at 37°C with 250 ng supercoiled pHOT1 plasmid substrate from a commercial topoisomerase 1 assay kit (Topogen Inc., Buena Vista, CO, United States). Bands were visualized after electrophoresis for 2 h at 70V on 1% (^w^/_v_) agarose with 0.01% (^w^/_v_) SDS and staining with GelRed (Biotium, Fremont, CA, United States) for 30 min.

### Assay of topoisomerase 1 inhibition in cells

HT-29 cells at ∼60% of confluent density were treated with apigenin or SN38 for 48 h. Cells were then rinsed with PBS, released by trypsinisation, and pelleted by centrifugation (200 x *g*, 10 min, 4°C). The pellet (∼10^7^ cells) was washed 3 times with cold PBS and resuspended in 180 μl ice-cold low-salt buffer (20 mM Tris-HCl, pH7.5, containing 5 mM KCl, 1 mM dithiothreitol, 10% (^v^/_v_) glycerol) and 1x HALT^®^ (Pierce Biotechnology, Rockford, IL, United States) protease inhibitors for a total of 10 min at 4°C and then physically disrupted by trituration 5x through a 27G needle. After further incubation for 30 min at 4°C, the homogenate was centrifuged (15,000 x *g*, 3 min, 4°C) and the nuclear pellet resuspended in 180 μl ice-cold high-salt buffer (low-salt buffer supplemented with 355 mM KCl) for total of 80 min at 4°C. The sample was then recentrifuged (15,000 x *g*, 10 min, 4°C) and the supernatant containing extracted nuclear proteins assayed for topoisomerase activity. Topoisomerase 1 activity was assessed as described (Webb and Ebeler 2003; Nitiss et al., 2012) using 5 μg protein, 200 ng supercoiled pEGFP DNA plasmid (Clontech Laboratories Inc., Mountain View, CA, United States) and incubation for 10 min at 37°C.

### Silencing of topoisomerase 1 using siRNA transfection

Small interfering RNA (siRNA) transfection of HT-29 cells was carried out according to the manufacture’s instructions, using 3.0 μl/well siPORT™ *Amine* Transfection Agent (AM4503) in 24-well plates. *Silencer* ^®^ Select negative control #1 siRNA was used as a negative control. Cells were seeded in 10% NCS DMEM at a density of 240,000 cells/ml and were transfected with an optimized concentration of 7.5 nM, using validated silencer^®^ Select siRNA specific for Topo1. 24 h following seeding, cells received a medium change in order to reduce cellular cytotoxicity. 48 h following siRNA transfection (when the knock-down became established as evaluated through western blot), cells were treated with 60 µM apigenin or its equivalent DMSO control. To examine the effect of apigenin on Topo1 knocked-down cells, cell-surface CD26 levels were assessed through a radioimmunoassay 48 h following treatment; this corresponded to 96 h after transfection.

### Molecular docking studies

Molecular docking studies were carried out using the computational software Discovery Studio Structure Base Design (SBD) suite from BIOVIA v17.1.0.16143 (Dassault Systemes, France). Flavonoids apigenin, luteolin, kaempferol and genistein were built in 3D and subjected to energy minimization by 500 steps each of steepest descent and conjugate gradient minimization respectively (RMS gradient 0.1 kcal/mol), using the CHARMm force field, distance-dependent dielectric constant and SHAKE algorithm using the *small molecules* tool in SBD. The x-ray coordinates of human Topo1-DNA complex were obtained from PDB (pdb id: 1K4T), water molecules were removed and the protein configuration was prepared using the *macromolecules* tool in SBD. The bound ligand topotecan was then used to create a 10 Å sphere to define the active site and the ligand (both cyclic lactone and open carboxylate) was deleted. In the next step, docking studies of flavonoids were carried out using the CDOCKER algorithm which is based on a simulated-annealing protocol using the CHARMm force field, 2,000 heating steps, 700 K target temperature and 5,000 cooling steps with a target temperature of 300 K to obtain 10 docked ligand poses with the Topo1-DNA complex. The binding poses were ranked using CDOCKER energy in kcal/mol, by determining the polar and non-polar interactions and bond distance parameters.

### Statistical analysis

Unless otherwise noted, figures are representative of independent experiments conducted on at least three separate occasions. Statistical analyses were performed using Prism 8.0 software (GraphPad, San Diego, CA, United States). Comparisons of data were performed using two-way ANOVA with Bonferroni’s comparison test to compare the replicate means unless otherwise indicated. For all analyses a *p*-value <0.05 was considered as the minimum for statistical significance.

## Results

### Other inhibitors of CK2 do not reproduce the ability of apigenin to interact with irinotecan

In our original work showing that apigenin can upregulate the protein CD26 on human colon carcinoma cells we noted that apigenin had a unique interaction with the anticancer drug irinotecan (Lefort and Blay 2011). We have since documented how multiple chemotherapeutic agents have the additional property, independent of their cytotoxic action, of upregulating CD26 (Cutler et al., 2015). However while the effects of combining apigenin with, for example, 5-fluorouracil or oxaliplatin are no more than additive, the interaction with irinotecan is highly synergistic.

Irinotecan enhances the *potency* for apigenin to increase CD26 by 30-fold (Lefort and Blay 2011). Conversely, apigenin itself enhances the *potency* of the irinotecan effect; it leads to a 4.2-fold enhancement of irinotecan potency, with 30 µM apigenin causing a reduction in the irinotecan EC_50_ from 4.7 μg/ml to 1.1 μg/ml (Lefort and Blay 2011).

We have recently shown (Lefort et al., 2020) that the ability of apigenin to elevate CD26 involves and requires inhibition of the activity of the kinase CK2. Furthermore, multiple synthetic CK2 inhibitors (Emodin, 6-methyl-1,3,8-trihydroxyanthraquinone; TBB, 4,5,6,7-tetrabromobenzotriazole; and DRB, 5,6-dichlorobenzimidazole 1-β-d-ribofuranoside) also upregulate CD26 (Lefort et al., 2020). We first examine whether CK2 inhibition is also necessary and sufficient for the ability to interact synergistically with irinotecan.

**FIGURE 1:**
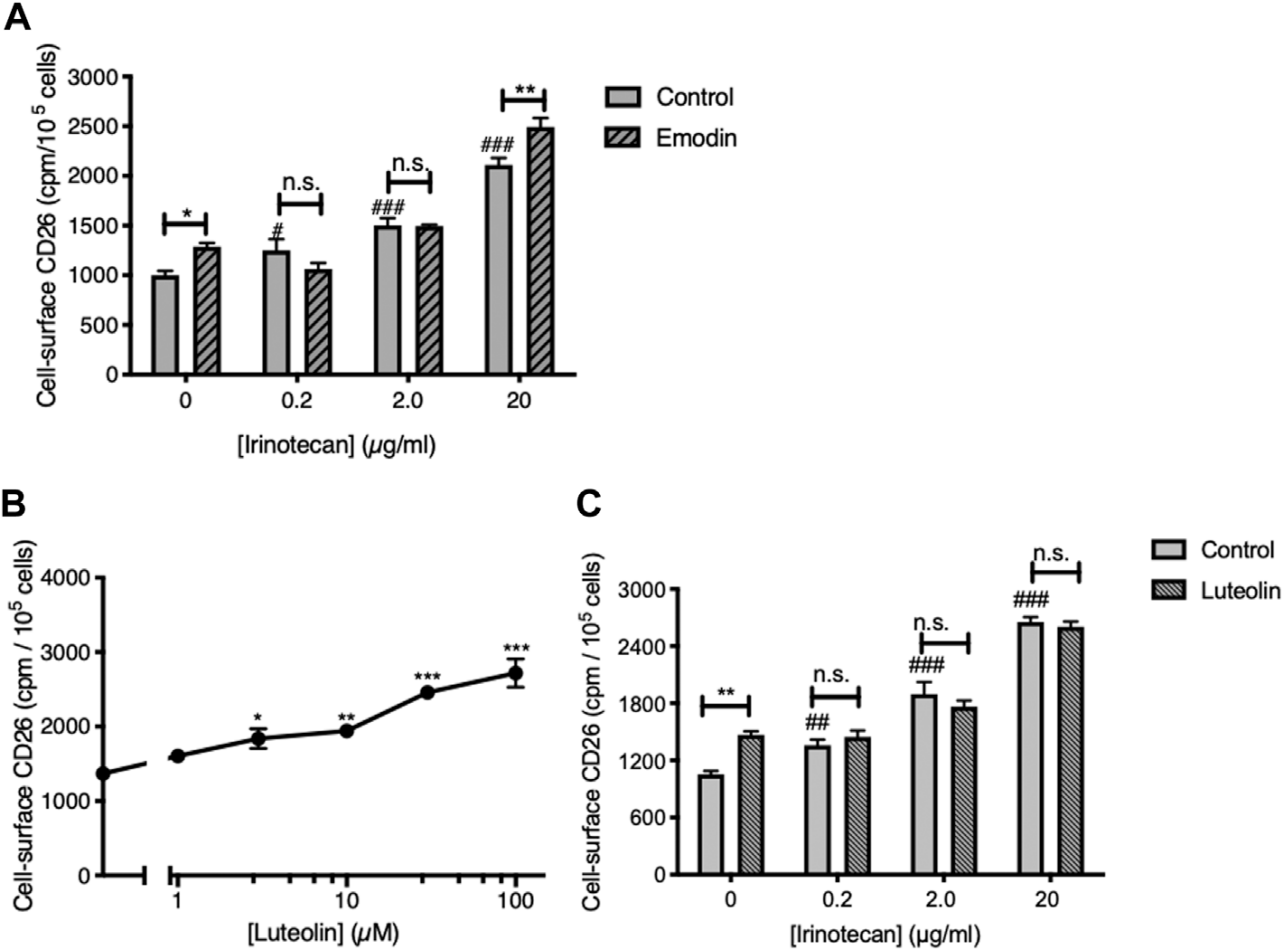
Neither CK2 inhibition, nor use of the apigenin metabolite luteolin, reproduce the interaction with apigenin seen with irinotecan. **(A)** The CK2 inhibitor emodin does not enhance the upregulation of CD26 by irinotecan. HT-29 cells were treated with irinotecan in the absence or presence of emodin (20 µM) as indicated, and cell-surface CD26 was assessed 48 h afterward. Data are means ± SEM (*n* = 4); *p < 0.05, **p < 0.01, enhancement by emodin; n.s., not significant; #*p* < 0.05 and ###*p* < 0.001 enhancement by irinotecan. **(B)** Luteolin enhances cell-surface CD26 in a dose-dependent manner. Means ± SEM (*n* = 4); *p < 0.05, **p < 0.01 and ***p < 0.001. **(C)** Luteolin does not enhance the upregulation of CD26 by irinotecan. Means ± SEM (*n* = 4); **p < 0.01, enhancement by luteolin (30 µM); n.s., not significant; ##*p* < 0.01 and ###*p* < 0.001 enhancement by irinotecan.

Consistent with our earlier work (Lefort et al., 2020) the CK2 inhibitor emodin was itself able to elevate cell-surface CD26 (Figure 1A). Furthermore, the apigenin metabolite luteolin (3′,4′,5,7-tetrahydroxyflavone), which is also an inhibitor of CK2 (Li et al., 2009; Lolli et al., 2012) was itself able to upregulate CD26 in a dose-dependent fashion (Figures 1B, C). As well, treating the cells with irinotecan produced a dose-dependent elevation of CD26 (Figures 1A, C) comparable to that we had observed before (Lefort and Blay 2011). However, neither emodin nor luteolin showed an ability to interact productively with irinotecan, nor the ability to increase the potency of irinotecan to act on CD26 (Figures 1A, C). Indeed, with the single exception of emodin at the highest dose of irinotecan, the effect of irinotecan was to mask the positive effect of the CK2 inhibitors.

Therefore, we conclude that although CK2 kinase inhibition is a key step in allowing flavonoids to elevate CD26, it is not sufficient to generate a synergism with irinotecan. Furthermore, this aspect of apigenin action has structural specificity in that it is not shared with its metabolite luteolin, which differs from apigenin only in the addition of a further hydroxyl group in the 3′ ring position. Apigenin also is more efficaceous in elevating CD26 and its associated activities than its isoflavone equivalent genistein or kaempferol, an analogue with an additional hydroxyl in the 3 position (Lefort and Blay 2011).

### The apigenin interaction with irinotecan is not seen with topoisomerase 2 inhibitors

**FIGURE 2:**
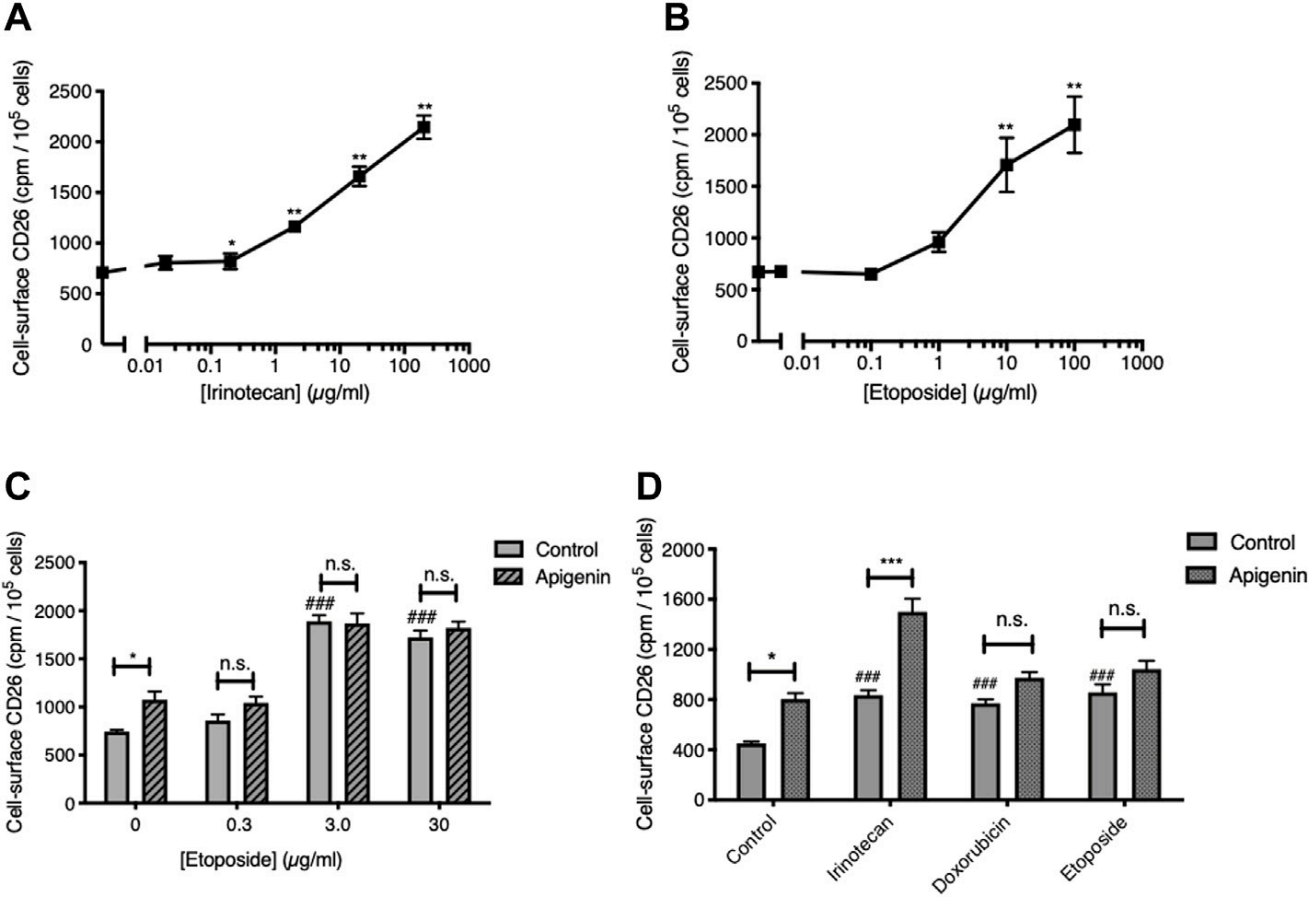
Topoisomerase 2 inhibitors elevate CD26 but do not reproduce the interaction with apigenin. **(A,B)** Topoisomerase 1 and 2 inhibitors both enhance cell-surface CD26. HT-29 cells treated for 48 h. Means ± SE (*n* = 4), one-way ANOVA followed by Dunnett’s test; *p < 0.05 and **p < 0.01, enhancement in CD26 by topoisomerase inhibitors. **(C)** Apigenin does not enhance the action or potency of etoposide. Means + SE (*n* = 4); *p < 0.05, enhancement by apigenin (30 µM); ###*p* < 0.001 up-regulation by etoposide. **(D)** Apigenin interacts solely with the Topo1 inhibitor irinotecan. Means + SE (*n* = 4); *p < 0.05, ***p < 0.01 enhancement by apigenin (30 µM); ###*p* < 0.001 up-regulation by topoisomerase inhibitors. Concentrations are: Irinotecan, 2 μg/ml; doxorubicin, 1 μg/ml; etoposide, 10 μg/m.

We next asked if this interaction would be present if apigenin were combined with type 2 topoisomerase-targeted anticancer drugs. As anticipated, given the breadth of chemotherapeutic triggers to this response, Topo2-active agents were able to elevate the level of CD26 on viable CRC cells (Figure 2). The response to the standard Topo2 drug etoposide was quantitatively similar to that for irinotecan (Figures 2A, B), and CD26 upregulation was also seen with another such agent, doxorubicin (Figure 2D). However, neither etoposide nor doxorubicin gave a further elevation of CD26 when combined with apigenin, unlike irinotecan (Figures 2C, D). Indeed, the response to apigenin was reduced to below statistical significance in the presence of both Topo2 inibitors. This is in agreement with our prior findings (Lefort and Blay 2011) that the synergistic potential of apigenin in elevating CD26 is restricted to the agent irinotecan, of the broad variety of anticancer drugs from different classes of activity, which now includes 5-FU, oxaliplatin, and irinotecan (Lefort and Blay 2011), cisplatin, vinblastine, methotrexate (Cutler et al., 2015) and etoposide and doxorubicin (this study).

### The ability of apigenin to upregulate CD26 involves Topo1 but is not due to altered Topo1 expression

**FIGURE 3:**
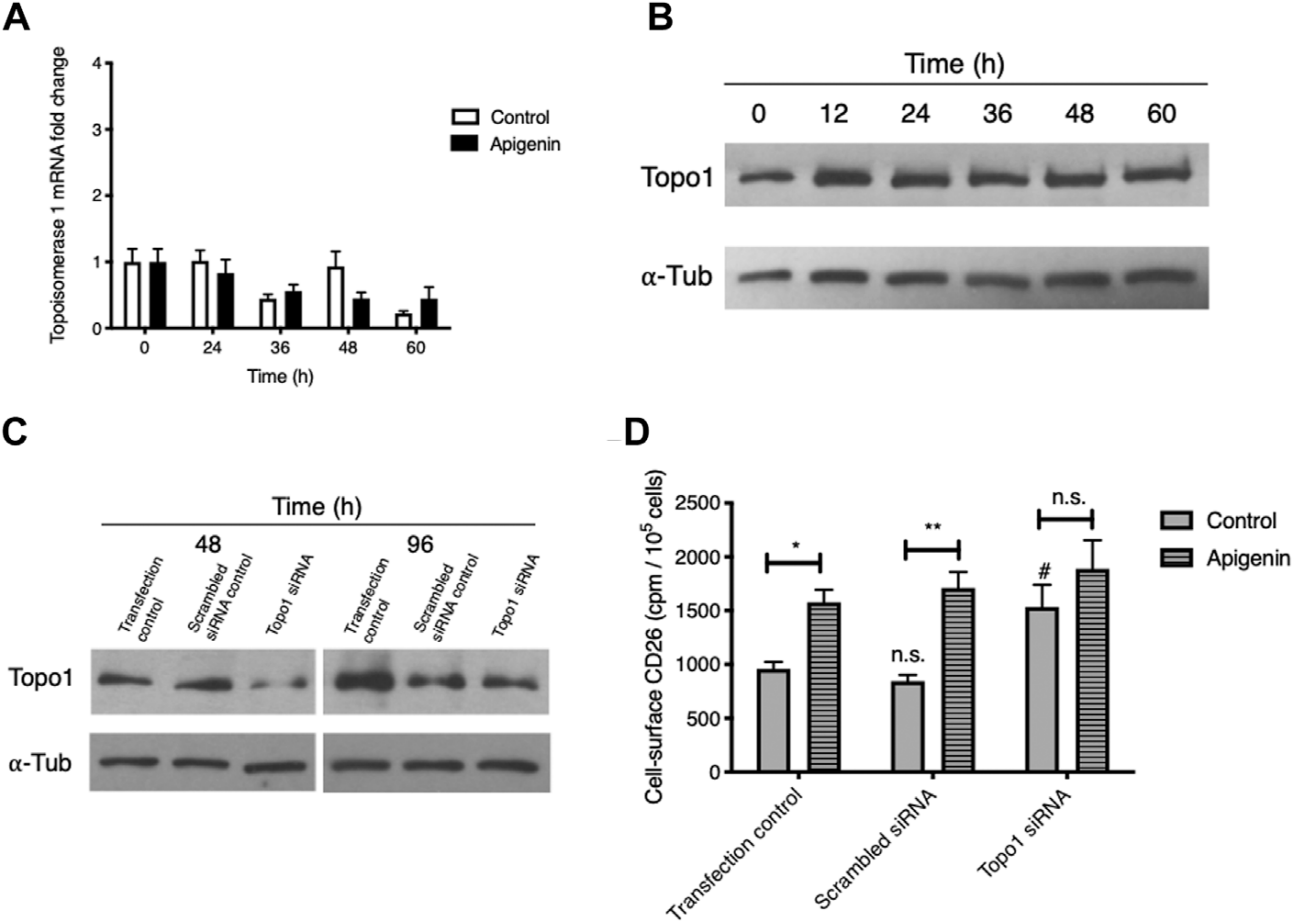
Apigenin’s ability to upregulate CD26 requires Topo1 but does not come from changes in Topo1 expression. **(A)** Apigenin does not alter *Topo1* mRNA expression. HT-29 cells were treated with apigenin (30 µM) or vehicle control for the times indicated and *Topo1* mRNA levels were quantified by q-RT-PCR. Values are means ± SEM (*n* = 3). **(B)** Apigenin does not alter Topo1 protein expression. HT-29 cells were treated with apigenin (60 µM) for the times indicated and Topo1 protein levels were quantified by western blotting relative to a-tubulin as loading control. **(C)** siRNA knock-down of Topo1. Topo1 protein levels were measured after 48 h or 96 h by western blot. **(D)** Knock-down of Topo1 enhances cell-surface CD26 and abrogates the apigenin upregulation of CD26. CD26 levels were evaluated 48 h following apigenin (60 µM) treatment. Values are means ± SEM (*n* = 4), from 3 independent experiments. #, *p* < 0.05, enhancement by knock-down; *, *p* < 0.05 and **, *p* < 0.01, enhancement by apigenin. n.s., not significant.

*Topo1* mRNA expression showed some variation over a 60-h time course (Figure 3A), likely reflecting cell cycle-dependent changes as we have noted for chemokine receptor CXCR4 (Richard et al., 2006). Apigenin treatment led to a modified time profile but no aggregate change in *Topo1* expression over the entire experimental period. The abundance of cellular Topo1 protein similarly showed no change relative to α–tubulin (Figure 3B). Apigenin does not therefore substantially alter the amount of Topo1 mRNA or protein.

To further probe for the importance of Topo1 in apigenin action, we knocked down expression using an siRNA approach with a combination of 3 validated siRNA constructs, and then subsequently tested the cells to see whether apigenin would still enhance CD26 levels. We were able to substantially (> 75%) knock down Topo1 protein at 48 h (Figure 3C) while retaining good cellular viability, which is also taken into account in our CD26 radioimmunoassay. The reduction in Topo1 was largely preserved at 96 h, which represents the total period covered for apigenin treatment and CD26 immunoassay (Figure 3C).

As shown in Figure 3D, the sham-transfected cells retained a robust apigenin response for the elevation of CD26 (mean increase, 114%). The baseline CD26 at the cell surface, and the ability to be upregulated by apigenin, were unaltered using a scrambled siRNA negative control. However, with Topo1 knockdown (i) the control-treated cells showed a significant increase in CD26, and (ii) the apigenin-treated cells showed no increase in CD26 above sham and scrambled controls that had received apigenin, and had lost their response to apigenin relative to the appropriate knock-down control (Figure 3D). This provides direct evidence for the involvement of Topo1 activity in the CD26 upregulatory response, and suggests that apigenin’s effect may depend on functional Topo1.

### Apigenin directly suppresses topoisomerase 1 activity

The evidence that apigenin might act through Topo1, and the unique interaction with the Topo1 poison irinotecan, suggest that apigenin might affect topoisomerase 1 activity. We assessed this directly in both whole cells and an *in vitro* enzyme system.

**FIGURE 4:**
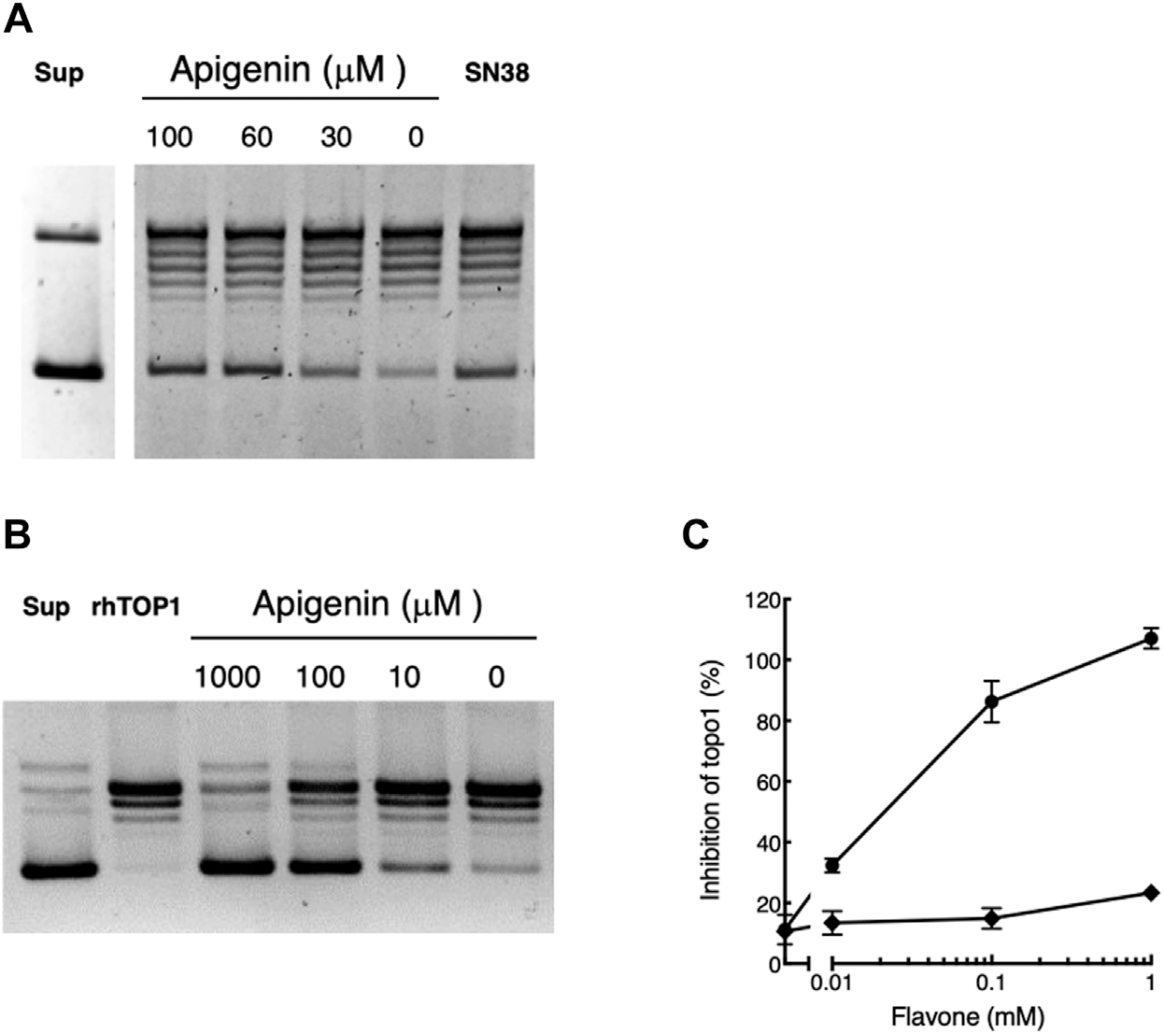
Apigenin suppresses topoisomerase 1 activity. **(A)** Apigenin suppresses topoisomerase 1 activity in whole cells. HT-29 cells were treated with apigenin for 48 h at the concentrations indicated. The original supercoiled DNA is shown (‘Sup’) as well as the effect of treating cells with 30 nM SN38 (positive control for Topo1 inhibition). The lowest band is the supercoiled DNA that is successively uncoiled (ladder) by cellular Topo1 activity to yield the final uncoiled structure (topmost band). Maximal uncoiling is present with untreated cells (“0 µM apigenin”) and this is inhibited by increasing concentrations of apigenin or exposure to SN38 (more intense lowest band). Representative of three independent experiments. **(B)** Representative result showing the gel separation of supercoiled plasmid (“Sup”), the effect of incubation with rhTopo1 alone and the result of co-incubation with apigenin at concentrations shown. As for panel A the lowest band is input supercoiled DNA that shows uncoiling to higher bands in the presence of Topo1, in this case rhTopo1. Increasing concentrations of apigenin added directly to the reaction mixture inhibit Topo1 activity, such that at 1,000 µM apigenin the uncoiling activity of the enzyme is essentially blocked. **(C)** rhTopo1 activity was measured in the presence of apigenin (•) or luteolin (◆) at the concentrations shown. Representative of three independent experiments.

We first tested whether apigenin treatment led to inhibition of the topoisomerase 1 activity isolated from pretreated whole cells. As illustrated in Figure 4A recoverable Topo1 leads to progressive uncoiling of supercoiled plasmid DNA (‘Sup’) to produce a series of products culminating in the fully relaxed and slowest-migrating (topmost) band. Treating the cells for 48 h with apigenin over its active concentration range 0 µM–100 µM led to progressive inhibition of the topoisomerase activity in those cells with greater preservation of supercoiled structure on the final assay. Maximal effect in this system was at 60 μM apigenin, consistent with its effect in elevating cellular CD26. The efficacy of apigenin was equivalent to that of a maximally effective dose (30 nM) of the irinotecan active metabolite SN38, which is a cell-permeable direct inhibitor of Topo1 (Cutler et al., 2015) (Figure 4A). The inhibitory effect of apigenin was observable at culture confluencies between 30% and post-confluent, and for cultures with apigenin treatment times of 6 h–48 h (data not shown).

When apigenin was added directly into an assay of topoisomerase 1 activity using purified rhTopo1, it substantially inhibited the uncoiling of supercoiled DNA (Figure 4B). Inhibition was substantial (greater than 30%) with 10 µM apigenin (Figure 4C), which is below the EC_50_ of 32.8 µM ± 1.1 µM seen in whole cells (Lefort and Blay 2011). Inhibition of activity exceeded 80% with apigenin present at 100 μM, the highest exposure to which whole cells may be exposed without substantial acute toxicity (Lefort and Blay 2011), and was essentially complete (3 independent experiments) with apigenin present at 1 mM. The solvent vehicle, DMSO, slightly interfered with Topo1 activity (Figure 4C), but this did not exceed 10% inhibition at the highest concentration (1.7%^v^/_v_) used in these assays.

Luteolin, over the concentration range 0 µM–100 µM, within which it is able to elevate CD26 levels in whole cell assays (Figure 1B) did not directly inhibit Topo1 in these assays (Figure 4C). It had a minor (< 15%) effect at the highest concentration used (1 mM), which exceeds that attainable with whole cells.

Apigenin therefore: (i) inhibits Topo1 activity in whole cells over the same concentration range at which it elevates CD26, (ii) inhibition is present over the time course in which it produces progressive elevation of CD26 (Lefort and Blay 2011), (iii) it inhibits Topo1 activity directly in an *in vitro* assay with recombinant enzyme, (iv) luteolin the predominant metabolite of apigenin, which is also able to elevate CD26 but does *not* exhibit synergy with irinotecan (SN-38) in this response, is *unable* to inhibit the activity of Topo1. We therefore propose that the ability of apigenin to interact synergistically with irinotecan in causing elevation of cell-surface CD26 is due to direct interaction with Topo1 enzyme.

### Apigenin has a direct fit into the Topo1-DNA complex

We propose that Topo1 is one of the components of the pathway(s) that cause cells to express increased amounts of CD26 on their cell surface, and that apigenin has a particular fit to Topo1 that enables its unique interaction with irinotecan. We used computational modeling of binding interactions to assess the feasibility of this proposal.

The binding interactions of apigenin, luteolin, kaempferol and genistein with human DNA-topoisomerase 1 (Topo1) complex were investigated by molecular docking studies. The x-ray crystal structure of human Topo1-DNA in complex with its known inhibitor topotecan [pdb id: 1K4T; (Staker et al., 2002)] was used as the basis for these studies.

**FIGURE 5:**
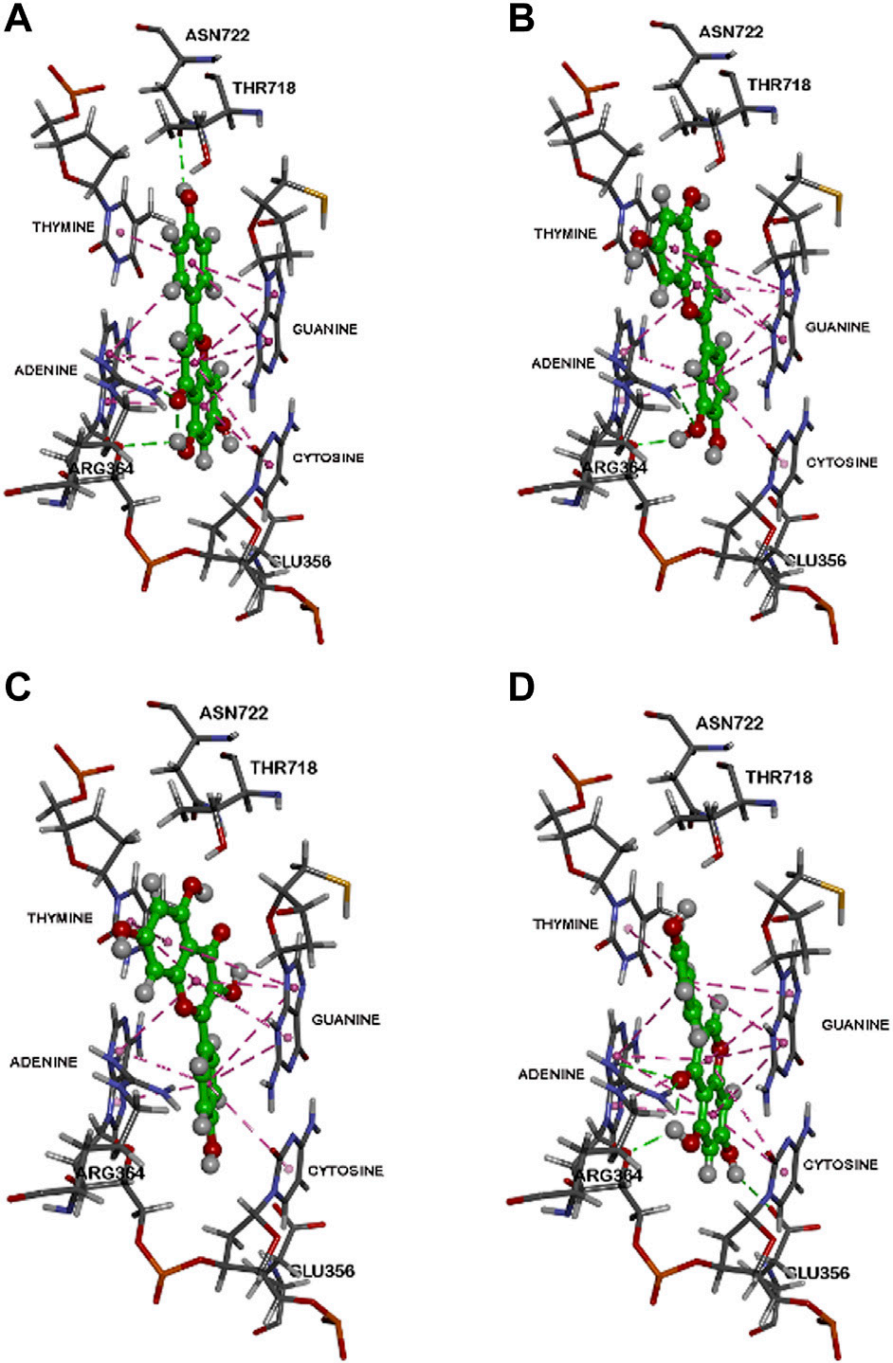
Binding modes of flavonoids within the human Top 1-DNA complex. The binding modes within the human Topo1-DNA complex (pdb id: 1K4T) are shown for, **(A)** apigenin, **(B)** luteolin, **(C)** kaempferol, and **(D)** genistein. Polar and non-polar interactions are colored coded and details are provided in text.

**FIGURE 6:**
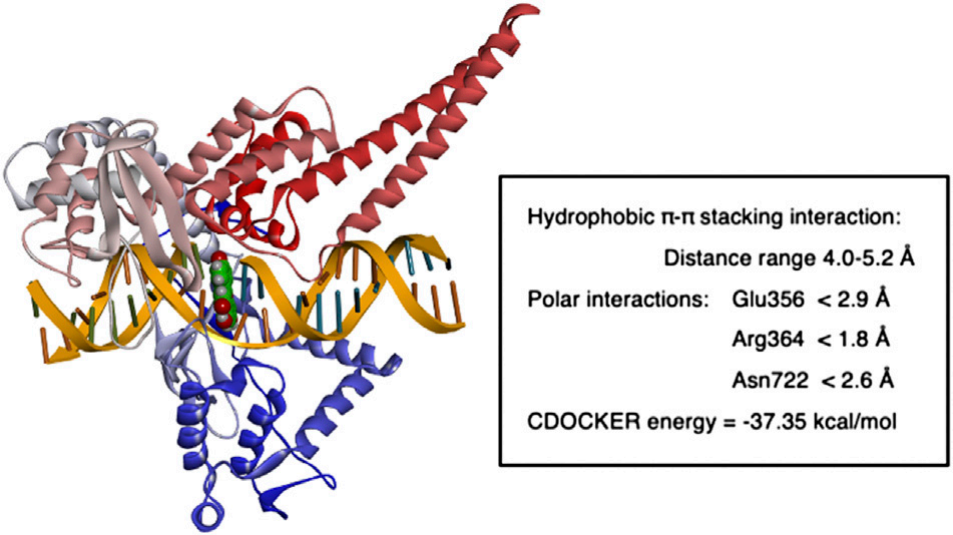
Molecular modeling of apigenin fit within in human Top 1-DNA complex. The figure shows the result of computational modeling for apigenin bound within the Topo1-DNA complex and key interaction distances.

Computational analysis of molecular interaction with the Topo1-DNA complex showed that each of these flavones in planar conformation is able to intercalate at the site of DNA cleavage (Figures 5, 6).

For apigenin (4′,5,7-trihydroxyflavone, Figure 5A) after intercalation the flavone structure undergoes several hydrophobic π-π stacking interactions with the DNA base pairs adenine, guanine, thymine and cytosine (distance range = 4.0 Å–5.2 Å). Furthermore, bound apigenin is in contact with the Topo1 enzyme *via* polar interactions, with the C7 OH undergoing a hydrogen bond with the Glu356 side chain (distance <2.9 Å), the benzopyran-4-one ketone forming a hydrogen bonding interaction with the Arg364 side chain (distance <1.8 Å) and the C2 phenolic group undergoing hydrogen bonding interaction with the side chain of Asn722 (distance <2.6 Å), as shown in Figure 5A.

Luteolin (3′,4′,5,7-tetrahydroxyflavone, Figure 5B) is the 3′-hydroxylated metabolite of apigenin. Modeling luteolin in the Topo1-DNA complex showed that it is also able to intercalate between DNA base pairs. Compared to apigenin, it exhibits a completely different binding mode where the planar benzopyran-4-one ring is primarily interacting with guanine and thymine base pairs on either sides instead of adenine and cytosine (distance <5.5 Å). These π-π stacking interactions lead to a more stable complex (CDOCKER energy = ‒42.02 kcal/mol) compared to apigenin (CDOCKER energy = ‒37.35 kcal/mol). In addition, luteolin was also in contact with amino acids of Topo1 *via* hydrogen bonding interactions (Glu356, Arg364, and Asn722; distance <2.8 Å) as shown in Figure 5B.

Kaempferol (3,4′,5,7-tetrahydroxyflavone), which differs from apigenin in the addition of a hydroxyl at the 3 position, exhibited a similar binding mode to luteolin in the Topo1-DNA complex (Figure 5C). It was able to intercalate between DNA base pairs by several π-π stacking interactions and also formed hydrogen bonding interactions with Topo1 amino acid residues Arg364 and Asn722. The ternary complex energy (CDOCKER energy = ‒38.11 kcal/mol) showed that it was able to exhibit slightly superior binding compared to apigenin.

The flavonoid genistein (4′,5,7-trihydroxyisoflavone), which is apigenin’s isoflavone analogue, shows a similar binding mode to apigenin (Figure 5D) in which the benzopyran-4-one is flanked by guanine, adenine and cytosine *via* π-π stacking interactions. Genistein also undergoes additional polar interactions with Topo1 amino acids Glu356, Arg364, and Thr718 (distance <3.0 Å). Genistein exhibited a less stable ternary complex than the other flavones (CDOCKER energy = ‒30.69 kcal/mol).

The fit of apigenin into the topotecan-stabilized Topo1-DNA complex, and key interaction distances, are summarized in Figure 6. The molecular modeling studies show that all of these flavones potentially act as DNA base pair mimics, and that their planar conformation enables them to intercalate at the site of DNA cleavage. In this way they are similar to other natural products with planar structures that are able to intercalate into the DNA helix and have been found to inhibit the activities of both types 1 and 2 topoisomerases. It may be that apigenin’s action is like that of topotecan (Staker et al., 2002) and that it can bind and stabilize the Topo1-DNA complex to prevent DNA religation (Drwal et al., 2011; Jadaun et al., 2017). However, the affinity of binding under these conditions may be less important to the effect on Topo1 activity than the nature of the specific interactions with particular base pair combinations and/or Topo1 amino acid residue side chains. Our data show that luteolin, which has the greatest overall binding potency, does not reproducibly inhibit Topo1 activity to a substantial degree.

## Discussion

The ability to elevate cell-surface CD26 is a feature of many chemotherapeutic agents, including 5-fluorouracil, cisplatin, vinblastine, methotrexate, oxaliplatin and irinotecan (Lefort and Blay 2011; Cutler et al., 2015), etoposide and doxorubicin (this paper). It is also seen to different degrees extent with flavones such as apigenin, genistein, kaempferol (Lefort and Blay 2011) and luteolin (this paper). Transduction of the enabling signal involves a network of protein kinases, including CK2 (Lefort et al., 2020) and MAPK (Lefort and Blay, unpublished data) kinases. We have though noted a pharmacologic interaction only between apigenin and irinotecan, a chemotherapeutic drug that acts as a topoisomerase 1 inhibitor. Taken together, our findings here provide evidence for a unique involvement of the irinotecan (SN38) target Topo1 in CD26 upregulation by apigenin.

We have previously observed that multiple chemotherapeutic drugs have the ability to upregulate CD26 over a prolonged period (beginning 24 h–72 h after initial exposure), at doses below those causing cytotoxicity (Lefort and Blay 2011; Cutler et al., 2015). Our data here confirm that irinotecan, etoposide and doxorubicin have this action in HT-29 cells, producing elevations in cell-surface CD26 within the range 71%–212%. This phenomenon is seen in cells from different cancers including prostate, breast, lung and colon and is consistently demonstrable in different cell lines from a given cancer (Lefort and Blay 2011; Cutler et al., 2015). The increased abundance of CD26 is found at the cell surface and accompanied by elevations in dipeptidyl peptidase IV (DPP4) and adenosine deaminase (ADA)-binding activities, which are the functionalities of the CD26 protein (Lefort and Blay 2011; Cutler et al., 2015). As these functions serve to dampen external pathways that facilitate tumour progression, we have proposed that is an additional beneficial action of anticancer drugs due to the activation of antimetastatic pathways secondary to their cytotoxic action (Cutler et al., 2015).

Separately, we have shown that apigenin increases cell-surface CD26 on CRC cancer cells (Lefort and Blay 2011). This is common to relatively differentiated CRC cell lines (HT-29, HRT-18 and Caco-2) but not seen in less-differentiated cells such as those of the HCT-116, SW480 and SW620 lines, which express very low intrinsic levels of CD26 (Lefort et al., 2020). The apigenin enhancement of CD26 in CRC cells is independent of detectable cytotoxicity, but is accompanied by subsequent decreases in cell growth and profileration as measured by assays of DNA synthesis, energy metabolism and cell population number (Lefort and Blay 2011). We have also shown that elevation of CD26 by apigenin is preceded by inhibition of the activity of the kinase CK2 (Lefort et al., 2020). The cellular distribution of CK2 in a cancer cell differs from that of a normal cell. In the former, CK2 is mainly localized in a diffuse pattern in the nucleus and the cytoplasm whereas in a cancer cell, CK2 levels are higher in the nuclear compartment (Faust et al., 1999; Laramas et al., 2007). This raises the possibility that apigenin’s actions on CK2 might directly interplay with its ability to target Topo1 as reported here.

Topo1 plays a role in the pathway to elevate CD26. In addition to the ability of the Topo1 inhibitors irinotecan and SN38 to upregulate CD26, we show here that direct knockdown of Topo1 with a specific siRNA leads to a substantial (mean, 96.5%) increase in CD26. Moreover, there is a unique interaction with apigenin. Our molecular modeling shows that all 4 related flavones tested, apigenin, luteolin, kaempferol, and genistein, have the capacity to bind within the Topo1-DNA complex, but that the binding of apigenin is unique in terms of its angle relative to the base-pair structure, potentially giving apigenin the ability to interfere with topoisomerase function. Direct measurements of topoisomerase activities, both in whole cells and with purified recombinant Topo1, showed that apigenin has the ability to directly inhibit Topo1 activity, and that this occurs within the concentration range that is non-toxic and able to elevate CD26 on whole cells.

Although luteolin, the primary hydroxylated metabolite of apigenin, is itself able to elevate CD26, it does not inhibit topoisomerase activity (Figure 4C) and does not yield a synergistic effect with irinotecan (Figure 1C). These two actions are unique to apigenin. We propose that the unique ability of apigenin to sensitize cells to irinotecan (4.2-fold reduction in the EC_50_, (Lefort and Blay 2011)) and conversely that of irinotecan to increase sensitivity to apigenin (29.8-fold reduction in EC_50_, (Lefort and Blay 2011)) follow from direct cooperation of apigenin and the active metabolite of irinotecan SN-38 in modulating the activity of Topo1.

We conclude that apigenin has a unique fit into the Topo1-DNA functional complex that leads to direct inhibition of Topo1 activity, and suggest that this is the basis for the unique interaction with the CRC drug irinotecan. The combined action of these two agents in elevating cell-surface CD26, which may play a role in limiting local signals that facilitate tumour progression, may allow an approach to enhance the beneficial action of irinotecan in CRC, independently of its direct cytotoxic action. This is consistent with findings that irinotecan analogues may have beneficial clinical actions in cancer patients that are independent of their ability to inhibit Topo1 (Li et al., 2017).

## Data Availability

The original contributions presented in the study are included in the article/Supplementary Material, further inquiries can be directed to the corresponding author.
